# The Acupuncture Effect on Median Nerve Morphology in Patients with Carpal Tunnel Syndrome: An Ultrasonographic Study

**DOI:** 10.1155/2017/7420648

**Published:** 2017-06-06

**Authors:** Fatma Gülçin Ural, Gökhan Tuna Öztürk

**Affiliations:** ^1^Department of Physical Medicine and Rehabilitation, Yıldırım Beyazıt University Medical School, Ankara, Turkey; ^2^Ankara Physical Medicine and Rehabilitation Training and Research Hospital, Ankara, Turkey

## Abstract

**Introduction:**

The aim of this study was to explore the acupuncture effect on the cross-sectional area (CSA) of the median nerve at the wrist in patients with carpal tunnel syndrome (CTS) and, additionally, to identify whether clinical, electrophysiological, and ultrasonographic changes show any association.

**Methods:**

Forty-five limbs of 27 female patients were randomly divided into two groups (acupuncture and control). All patients used night wrist splint. The patients in the acupuncture group received additional acupuncture therapy. Visual analog scale (VAS), Duruöz Hand Index (DHI), Quick Disabilities of the Arm, Shoulder and Hand (DASH) questionnaire scores, electrophysiologic measurements, and median nerve CSAs were noted before and after the treatment in both groups.

**Results:**

VAS, DHI, Quick DASH scores, and electrophysiological measurements were improved in both groups. The median nerve CSA significantly decreased in the acupuncture group, whereas there was no change in the control group.

**Conclusion:**

After acupuncture therapy, the patients with CTS might have both clinical and morphological improvement.

## 1. Introduction

Carpal tunnel syndrome (CTS) is the most common local entrapment neuropathy resulting from compression of the median nerve as it passes through the carpal tunnel at the wrist. In the general population, CTS prevalence is about 1 to 5% and women are more affected than men [1, 2]. As CTS may be caused by the overuse of the hand, also some systemic conditions such as diabetes mellitus, rheumatoid arthritis, hypothyroidism, and pregnancy may be associated with this syndrome [3]. Pain, numbness, and tingling affecting first 3 to 4 fingers in the hand are the most common symptoms of CTS. Additionally, weakness and atrophy of the hand muscles, those innervated with the median nerve, may occur [4]. Clinical symptoms and electromyographic studies are useful for diagnosis. Rest, nonsteroidal anti-inflammatory drugs, splinting, corticosteroid injections, vitamin B6, physical therapy, and surgical procedures have been used for treatment [5]. In addition to these treatment techniques, acupuncture may be applied for treatment of patients with CTS. Acupuncture is a complementary and alternative medicine (CAM) method widely used in China and also western countries. Being a simple, inexpensive, and harmless treatment technique, it has been an accepted treatment modality for painful disorders.

Musculoskeletal ultrasound (US) has been increasingly used in physiatry practice in the last decade. In CTS, the median nerve can be enlarged with the increase in its cross-sectional area (CSA) in the wrist level due to swelling proximal to the entrapment site. US is an inexpensive and easily accessible imaging method and it can provide morphologic data of the median nerve in patients with CTS. Additionally, CSA of the median nerve was found to be associated with severity of CTS [6, 7]. A cut-off value of 9 mm^2^ for median nerve CSA at the wrist level was set with a high sensitivity (% 99) in CTS diagnosis [8]. Further, CSA of the median nerve at the wrist was a prognostic factor for carpal tunnel decompression surgery [9], and it might be used for monitoring of treatment [10]. In previous studies, acupuncture treatment has been shown as an effective and safe treatment modality for the CTS [11, 12]. However, to the best knowledge of the authors, the effect of the acupuncture on median nerve morphology has not been investigated in CTS patients before.

Accordingly, in this study, we aimed to evaluate the effect of acupuncture on median nerve CSA and to explore the correlation between clinical/electromyographic and ultrasonographic changes after acupuncture treatment in patients with CTS.

## 2. Methods

Twenty-seven female patients (45 limbs) with CTS were included in this randomized, controlled study. CTS diagnosis was made using electromyographic studies. The presence of radicular pain, polyneuropathy, radial or ulnar nerve diseases, severe CTS, history of trauma, and previous surgery of the hand and wrist were identified as exclusion criteria. The study protocol was approved by the local ethics committee. All subjects were informed about the study procedure and they provided written informed consent to participate.

Patients were randomly separated into two groups: acupuncture and control groups. All subjects were numbered according to the order of admittance to our clinic, and then randomization was performed via a computer program. Both extremities of bilateral CTS patients were included in the same group. All patients used night wrist splint for CTS (set at 0–5 degrees of wrist extension) for 4 weeks, while in the acupuncture group the patients received additional acupuncture treatment. Acupuncture was applied by the same experienced physician; during treatment, the patients were lying on the examination table in the supine position. Nine acupuncture points (PC-7, PC-4, PC-6, PC-8, HT-2, HT-7, HT-8, LU-9, and LI-11) which were described in detail previously were chosen for treatment [11, 13]. A 0.25 × 25 mm sized needle was placed vertically and kept for 25 minutes in these specific points. Acupuncture treatment was applied two or three days a week, for 4 weeks (a total of 10 sessions).

Age, body mass index (BMI), and disease duration of all patients were recorded. Severity of symptoms, hand function, and electrodiagnostic and US measurements were evaluated before and at the end of treatment. Severity of symptoms was measured by using the visual analog scale (VAS) (0–10 cm). Duruöz Hand Index (DHI) and Quick Disabilities of the Arm, Shoulder and Hand (DASH) scores were used to assess hand functions and disability. DHI consists of 18 questions evaluating activities requiring force, rotational motions, ability, precision, and flexibility of the first three fingers. Each question is scored between 0 and 5 (0: no symptoms; 5: impossible to do the activity). DHI score was calculated as the sum of the points [14]. Quick DASH consists of 11 questions evaluating disability of the upper limbs. Each question is scored between 1 and 5 (1: no difficulty during activity; 5: activity cannot be performed). To calculate the Quick DASH score, we subtracted 1 from the ratio of total points to answered questions and multiplied obtained values by 25 [15].

Electrophysiological tests were performed by using Nihon Cohden Neuropack (Tokyo, Japan) machine. Compound muscle action potential (CMAP) (normal > 6.8 mV), motor nerve conduction velocity (M-NCV) (normal > 49.4 m/sec), motor distal latency (normal < 3.8 msec), sensory nerve action potential (SNAP) (normal > 10 *μ*V), and sensory nerve conduction velocity (SNCV) (normal > 40.4 m/sec) were measured. CMAP was enrolled by using skin surface electrodes. The median nerve was stimulated at two different sites (wrist and elbow), and potentials were obtained from abductor pollicis brevis muscle. SNAP was recorded from the wrist via antidromic stimulation of the median nerve at the second finger. According to the electrodiagnostic tests, diagnosis of CTS was classified as mild, moderate, and severe (mild: reduced S-NCV; moderate: reduced S-NCV and prolonged motor distal latency; severe: absence of SNAP and/or reduced CMAP).

**Figure 1:**
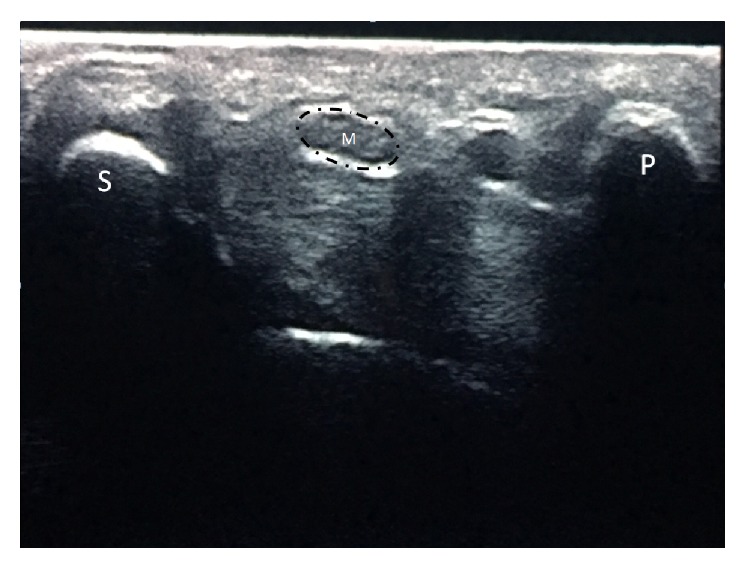
Measurement of median nerve cross-sectional area. M: median nerve; S: scaphoid; P: pisiform; dashed line: cross-sectional area.

CSA of the median nerve was measured by using US (MyLab Series; Esaote Biomedica, Italy) with a 6 to 12 MHz probe. During imaging, patients were seated; the shoulder was kept in neutral rotation, the elbow at 90° of flexion, and the forearm in supination position. CSA of the median nerve was measured at the proximal carpal tunnel during axial imaging. The scaphoid and pisiform bones were identified as bony landmarks for the proximal tunnel, where the CSA was measured (Figure 1).

For statistical analysis, SPSS (SPSS Inc., Chicago, Illinois, USA) version 22.0 program was used. Data were expressed as mean ± standard deviation. To compare demographic, clinical, electrodiagnostic, and US measurements between the two groups, Mann–Whitney *U* test or chi-square test was used (where appropriate). Comparison of clinical and electromyographic features and US measurements between pretreatment and posttreatment in each group was performed by using Wilcoxon signed-rank test. Correlations between clinical/electromyographic and US changes were analyzed by using Spearman correlation coefficients. The statistical significance was considered as *p* < 0.05.

## 3. Results

All patients completed the study. Demographic features of subjects are given in Table 1. The groups were similar with regard to age, BMI, duration of disease, and severity of CTS (*p* > 0.05 for all).

Clinical characteristics and electromyographic and US measurements are summarized in Table 2. The VAS, DHI, Quick DASH scores, CMAP, SNAP, and M-NCV values were improved in both groups (all *p* < 0.05). Additionally, motor distal latency (*p* = 0.03) and S-NCV (*p* < 0.001) were increased, and median nerve CSA (*p* < 0.001) was decreased (*p* = 0.001) in the acupuncture group, while these parameters did not change significantly in the control group (*p* > 0.05). Percentage changes of VAS, Quick DASH, DHI scores, S-NCV, and CSA of the median nerve were higher in the acupuncture group compared to the control group (*p* < 0.05 for all) (Table 3). Changes in clinical features and electromyographic measurements did not show a significant correlation with US measurements in both groups (all *p* > 0.05). The percent changes in the severity of CTS did not differ in both groups.

## 4. Discussion

The aim of this study was to explore whether acupuncture treatment had any additional effect on median nerve morphology in patients with CTS via US imaging. The results of this study showed that CSA of the median nerve decreased after acupuncture treatment. Additionally, symptom severity, hand functions, and electromyographic measurements improved in both groups. However, the improvements in VAS, DHI, Quick DASH, and S-NCV were significantly higher in the acupuncture group. The change in median nerve CSA did not correlate with clinical and electromyographic changes. While previous studies investigated the effect of acupuncture on clinical symptoms and electromyographic studies, to the best of our knowledge, its effect on median nerve morphology was not investigated before.

The mechanism of acupuncture effect on CTS has not been identified clearly yet. However, in recent studies, with the help of magnetic resonance imaging, it has been shown that acupuncture treatment might alter brain activity or regulate limbic system activity in patients with CTS [16–18]. Additionally, acupuncture has immune modulator and anti-inflammatory effects which might affect the inflammation in the entrapped median nerve in the carpal tunnel [19, 20]. In the previous literature, positive effects of acupuncture were shown in CTS patients [11, 12, 21]. Previously, in a randomized controlled study, acupuncture effects were compared with oral steroids in CTS patients [21]. It was shown that acupuncture treatment was superior in global symptom score, motor distal latency, and sensory distal latency compared to steroid injection during the evaluation at the end of 13 months. In a study including sixty-one CTS patients, 10-session acupuncture effect was compared with night splints [22]. At the end of 5 weeks, acupuncture has been found to be as effective as night splints on symptom severity and functional status. In another study, comparing an additional effect of 8-session acupuncture treatment together with night splints with splints alone, it was found that acupuncture treatment improved clinical symptoms and NCV. Consequently, the authors concluded that acupuncture may alleviate the subjective symptoms of CTS and could be considered in treatment programs of these patients [23]. Hadianfard et al. [11], reported that short-term acupuncture treatment is more effective than ibuprofen on clinical and electrophysiological findings of mild to moderate CTS. However, in a systematic review, it was stated that the evidence of acupuncture for CTS treatment was not satisfied because of poor methodological quality [24]. In our study, patients were evaluated at the end of the treatment, and when compared to the baseline values, reduction of symptoms and improvements of hand functions and electrophysiological findings were found to be higher in the acupuncture group than in the control group. Our findings were consistent with the previous studies. Further, in this study, we found that CSA of the median nerve was decreased in the acupuncture group. To the best of our knowledge, the effect of acupuncture on median nerve CSA in CTS patients was shown for the first time. According to our study results, acupuncture improves not only clinical and electrophysiological findings but also morphological features in patients with CTS.

Ultrasound measurement of median nerve CSA was used for diagnosis and assessment of response to therapy in patients with CTS [6–8, 10]. CSA greater than or equal to 9 mm^2^ may indicate the presence of CTS [8]. Additionally, it has been found that median nerve CSA at the wrist correlated with electrophysiological severity in CTS patients [6, 7]. In our study, although electrophysiological and morphological improvements were seen in the acupuncture group, the relationship between change of CSA and electrophysiological changes was not correlated. The small sample size of our study may cause this result.

The relatively small sample size and the lack of long-term monitoring of patients are major limitations of our study. Moreover, placebo effects of acupuncture cannot be evaluated. Sham acupuncture effect for CTS is debated. The effect of sham acupuncture in CTS in previous studies is conflicting. In a randomized controlled study, it was reported that acupuncture was not more effective than sham acupuncture when applied together with night splints [25]. In another study, acupuncture was found to be more effective than sham acupuncture in CTS patients [23]. In our study, sham acupuncture was not performed to the control group. Nevertheless, findings of our study are significant and noteworthy.

To summarize, in light of the results of the current study, we showed that acupuncture improves clinical and electrophysiological findings of CTS and also decreases CSA of the median nerve at the wrist. Our results need to be confirmed in future studies with larger sample, long-term monitoring, and placebo controlled studies. Finally, US seems to be a practical imaging tool for diagnosis and monitoring in these patients.

## Figures and Tables

**Table 1:** Demographic features of patients.

	Group A (*N* = 14 patients)	Group B (*N* = 13 patients)	*p*
	(25 limbs)	(20 limbs)	
Age (years)	50.5 ±6.1	51.5 ± 4.5	0.61
Body mass index (kg/m^2^)	29.7 ± 4.0	29.7 ± 2.4	0.84
Severity of CTS (mild/moderate)	9/16	7/13	0.90
Duration of CTS (months)	18.3 ± 6.6	19.3 ± 11.1	0.79

Data are given as mean ± standard deviation or *n*. CTS: carpal tunnel syndrome.

**Table 2:** Clinical characteristics and electromyographic and ultrasonographic measurements of patients.

	Group A (*N* = 25 limbs)			Group B (*N* = 20 limbs)		
	Before	After	*p*	Before	After	*p*
VAS	9.0 ± 1.0	4.8 ± 1.2	<**0.001**	9.2 ± 0.7	8.1 ± 1.2	**0.004**
Quick DASH	67.2 ± 9.6	56.8 ± 8.8	<**0.001**	69.9 ± 8.2	63.4 ± 6.8	**0.001**
Duruöz Hand Index	47.0 ± 10.4	37.0 ± 9.4	<**0.001**	62.2 ± 11.7	57.3 ± 12.1	<**0.001**
Motor nerve velocity (m/s)	57.4 ± 4.8	59.0 ± 4.2	**0.005**	55.8 ± 2.4	56.9 ± 2.3	**0.03**
Distal latency (ms)	4.3 ± 0.8	4.1 ± 0.8	**0.03**	4.0 ± 0.5	4.0 ± 0.6	0.90
Sensory nerve velocity (m/s)	31.0 ± 5.2	33.2 ± 5.4	<**0.001**	32.0 ± 4.3	32.6 ± 4.9	0.14
CMAP (mV)	12.9 ± 2.8	14.8 ± 3.4	<**0.001**	11.5 ± 2.2	12.3 ± 2.2	**0.04**
SNAP (*μ*V)	16.7 ± 5.2	17.6 ± 5.0	**0.03**	13.1 ± 4.9	14.4 ± 5.4	**0.01**
Median nerve CSA (mm)	11.6 ± 2.1	10.6 ± 1.8	<**0.001**	11.4 ± .1	11.3 ± 3.0	0.56

Data are given as mean ± standard deviation; VAS: visual analog scale; DASH: the Disabilities of the Arm, Shoulder and Hand; CSA: cross-sectional area; CMAP: compound muscle action potential; SNAP: sensory nerve action potential.

**Table 3:** Percent changes of clinical characteristics and electromyographic and ultrasonographic measurements.

	Group A (*N* = 25 limbs)	Group B (*N* = 20 limbs)	*p*
VAS	46.8 ± 10.7	11.4 ± 12.2	<**0.001**
Quick DASH	15.3 ± 6.5	9.0 ± 5.8	**0.002**
Duruöz Hand Index	21.7 ± 6.8	8.1 ± 5.2	<**0.001**
Motor velocity (m/s)	3.1 ± 4.7	2.1 ± 3.7	0.49
Distal latency (ms)	3.0 ± 6.4	0.2 ± 5.9	0.15
Sensory velocity (m/s)	7.3 ± 4.8	1.9 ± 5.1	<**0.001**
CMAP (mV)	15.4 ± 12.0	8.2 ± 15.2	0.09
SNAP (*μ*V)	7.1 ± 13.2	10.3 ± 14.4	**0.04**
Median nerve CSA (mm^2^)	8.1 ± 6.0	0.4 ± 7.3	<**0.001**

Data are given as mean ± standard deviation; VAS: visual analog scale; DASH: the Disabilities of the Arm, Shoulder and Hand; CSA: cross-sectional area; CMAP: compound muscle action potential; SNAP: sensory nerve action potential.
